# Berry Phenolic Antioxidants – Implications for Human Health?

**DOI:** 10.3389/fphar.2018.00078

**Published:** 2018-03-26

**Authors:** Beata Olas

**Affiliations:** Department of General Biochemistry, Faculty of Biology and Environmental Protection, University of Lodz, Lodz, Poland

**Keywords:** berries, phenolic compounds, antioxidants, health, oxidative stress

## Abstract

Antioxidants present in the diet may have a significant effect on the prophylaxis and progression of various diseases associated with oxidative stress. Berries contain a range of chemical compounds with antioxidant properties, including phenolic compounds. The aim of this review article is to provide an overview of the current knowledge of such phenolic antioxidants, and to discuss whether these compounds may always be natural gifts for human health, based on both *in vitro* and *in vivo* studies. It describes the antioxidant properties of fresh berries (including aronia berries, grapes, blueberries, sea buckthorn berries, strawberries and other berries) and their various products, especially juices and wines. Some papers report that these phenolic compounds may sometimes behave like prooxidants, and sometimes demonstrate both antioxidant and prooxidant activity, while others note they do not behave the same way *in vitro* and *in vivo*. However, no unwanted or toxic effects (i.e., chemical, hematological or urinary effect) have been associated with the consumption of berries or berry juices or other extracts, especially aronia berries and aronia products *in vivo*, and *in vitro*, which may suggest that the phenolic antioxidants found in berries are natural gifts for human health. However, the phenolic compound content of berries and berry products is not always well described, and further studies are required to determine the therapeutic doses of different berry products for use in future clinical studies. Moreover, further experiments are needed to understand the beneficial effects reported so far from the mechanistic point of view. Therefore, greater attention should be paid to the development of well-controlled and high-quality clinical studies in this area.

## Introduction

Natural phenolic compounds are found in many foods, including vegetables, fruits, tea, coffee, chocolate, wine, honey, and oil (Kulling and Rawel, 2008; Szajdek and Borowska, 2008; Chong et al., 2010; Chrubasik et al., 2010; Christaki, 2012; Kutlesa and Mrsic, 2016; Gomes-Rochette et al., 2016).

Recent years have seen increased consumption of berries, and fruit in general. Research suggests that this increased intake of fruits and berries may be associated with a reduced incidence of disorders induced by reactive oxygen species (ROS), including cardiovascular disorders, cancer and inflammatory processes (Gomes-Rochette et al., 2016). Berries and their products (i.e., berry juice and jam) are very often recognized as “superfoods.” They possess high concentrations of phenolic compounds, which have been found in *in vitro* and *in vivo* studies to possess a range of biological activities, including anticancer and antiplatelet activities, as well as antioxidant properties (Valcheva-Kuzmanova et al., 2006; Erlund et al., 2008; Kulling and Rawel, 2008; Szajdek and Borowska, 2008; Chong et al., 2010; Chrubasik et al., 2010; Christaki, 2012; Giampieri et al., 2012, 2015; McEwen, 2014; Nile and Park, 2014; Del Bo et al., 2015; Skrovankova et al., 2015; Wightman and Henberger, 2015; Kristo et al., 2016; Olas, 2016, 2017). However, these compounds may not influence the levels of oxidative stress biomarkers, and may even have prooxidative effects. In addition, the precise biological activities of berry phenolics are dependent on a range of factors including the class of phenolics, their concentration, the type of berry and even the form consumed, be it fresh berries, juice, wine, jam, oil or medicinal products. This review article summarizes the current knowledge concerning whether the phenolic compounds within berries may always have a beneficial influence on human health as antioxidants, and to what extent these compounds may sometimes act as prooxidants. The source information for this paper is derived not only from *in vitro* models, but also *in vivo* models.

## The Botanical Classification of Berries

Although, according to botanical terminology, a berry is a simple fruit with seeds and pulp produced from the ovary of a single flower with a fleshy pericarp, the term “berry” is also commonly used to refer in general to a small, pulpy and often edible fruit. Blueberries may be categorized as berries under both definitions, but grapes are berries only according to the botanical definition. Moreover, while strawberries and blackberries are typically referred to as berries, they are not officially categorized as such (Hickey and King, 2001).

Berries belong to several families, although the two key examples are the Rosaceae, including black chokeberry (*Aronia melanocarpa*), strawberry (*Fragaria ananassa*), red raspberry (*Rubus ideaus*), black raspberry (*Rubus occidentalis*), blackberry (*Rubus fruticosus*) and cloudberry (*Rubus chamaemorus*), and the Ericaceae, including cranberry (*Vaccinium macrocarpon*), bilberry (*Vaccinium myritillus*), lowbush blueberry (*Vaccinium angustifolium*), highbush blueberry (*Vaccinium corymbosum*). Examples of berries from other families include blackcurrants (*Ribes nigrum*; family: Grossulariaceae), sea buckthorn (*Elaaagnus rhamnoides* (L.); family: Elaeagnaceae) and grapes (*Vitis*; family: Vitaceae).

## The Chemical Composition of Berries

A huge variety of phenolic compounds are produced by plants, with 1000s recognized throughout the plant kingdom. They can be found in various parts of the plant, but particularly the fruits, leaves and seeds, where they are typically involved in the defense against ultraviolet radiation and pathogens. Phenolics possess one or more aromatic rings bearing one or more hydroxyl groups. They occur in free and conjugated forms with acids, sugars, or other water-soluble or fat-soluble compounds (Szajdek and Borowska, 2008; Nile and Park, 2014; Del Bo et al., 2015; Skrovankova et al., 2015).

For years, phenolic compounds were regarded as anti-nutritional compounds, and in some cases as toxic and mutagenic. Their anti-nutritional activities result from their interactions with proteins, which reduce nutrient assimilation by the inhibition of proteolytic, lipolytic and glycolytic enzymes. Moreover, metal cations are often made unobtainable by complexing with phenolic compounds in humans consuming a plant-based diet. It is important to note that the toxicity of phenolic compounds has not yet been fully recognized and was ignored for years (Bisson et al., 2015).

Berries are not only a source of non-nutritive compounds, including phenolics (Singh and Basu, 2102), but are also a rich source of wide variety of nutritive compounds, including sugars (glucose, fructose) and minerals (phosphorus, calcium, iron, potassium, magnesium, manganese, sodium and copper) (Kulling and Rawel, 2008; Szajdek and Borowska, 2008; Giampieri et al., 2012; Del Bo et al., 2015; Malinowska and Olas, 2016). In addition, iron and manganese are important components of antioxidant enzymes. Berries contain a large amount of the vitamins A, C and E, which act as antioxidants and may reduce the inflammation process (Skrovankova et al., 2015). Blackcurrants and sea buckthorn berries have particularly high concentrations of vitamin C, ranging from 120 to 215 mg per 100 g fruit for blackcurrants, and as high as 600 mg per 100 g fruit for sea buckthorn berries (Olas, 2016; Malinowska and Olas, 2016). Furthermore, berries contain low concentrations of lipids but high concentrations of dietary fiber, which has a nutritional function and reduces the level of low density lipoprotein (LDL) in serum. In addition, it is notable that sea buckthorn oil (extracted from seeds and fruits) and grape seed oil are rich source of fatty acids, unsaturated fatty acids in particular, which have beneficial effects on cardiovascular diseases, neurodegenerative diseases and cancer (Olas, 2016). All these compounds together have a synergistic and multifunctional effect on human health. The chemical composition of a particular berry depends on a range of factors, such as cultivar and variety, plant nutrition, time of harvest, growing location and environmental conditions (Skrovankova et al., 2015).

## The Chemical Structure of Phenolic Compounds With Antioxidant Properties

### Anthocyanins

Anthocyanins confer the blue, purple and red color of many fruits, including berries. However, berry anthocyanins are not only responsible for fruit color, but also may be used as natural pigments for the food industry (He and Giusti, 2009; Lee et al., 2015). In addition, anthocyanins are known to be one of the most powerful natural antioxidants. Berries are one of the richest sources of anthocyanins among all the fruits (He and Giusti, 2009; You et al., 2011; Lee et al., 2015; Olivas-Aguirre et al., 2016) and are found at the highest concentrations in the skins of berries. Anthocyanins consist of an aromatic ring bonded to a heterocyclic ring containing oxygen, which is also bonded by a carbon-carbon bond to a third aromatic ring. They can be classified into six forms based on the presence of hydroxyl and methoxyl substitutions on the B-ring: cyanidin, malvidin, peonidin, petunidin, pelargonidin and delphinidin (He and Giusti, 2009). The most common types of anthocyanins present in various berries are given in **Table 1**.

**Table 1:** Major types of anthocyanins, which are presented in various berries (Lee et al., 2015; Nayak et al., 2015; Wang et al., 2015; Kristo et al., 2016; Kšonžeková et al., 2016; Samoticha et al., 2017; modified).

Berries	Type of anthocyanins					
	
	Pelargonidin	Cyanidin	Delphinidin	Peonidin	Malvidin	Petunidin
Aronia berries (*Aronia melanocarpa*)		+ (major – cyaniding 3-galactoside, cyaniding 3-arabinoside; minor – cyaniding 3-glucoside; cyaniding 3-xyloside)				
Bilberries (*Vaccinium myritillus*)		+ (major – cyanidin-3-galactoside; minor – cyanidin glucoside; cyanidin arabinoside)	+ (minor – delphinidin arabinoside; delphinodin galactoside; delphinidin glucoside)	+ (minor – peonidin glucoside)	+ (minor – malvidin galactoside; malvidin arabinoside)	+ (minor – petunidin glucoside)
Blackcurrants (*Ribes nigrum*)		+ (major – cyaniding 3-rutinoside; minor – cyanidin 3-glucoside)	+ (major – delphinidin 3-glucoside, delphinidin 3-rutinoside)			
Blackberries (*Rubus fruticosus*)		+ (major – cyanidin-3-glucoside; minor – cyanidin-3-rutinoside, cyanidin-3-dioxalylglucoside, cyanidin-3-xyloside; cyanidin-3-malonylglucoside)				
Blueberries (*Vaccinium corymbosum*)		+ (major – cyaniding 3-galactoside; minor – cyaniding 3-glucoside, cyaniding 3-arabinoside)	+ (major – delphinidin 3-galactoside, delphinidin 3-arabinoside; minor – delphinidin 3-glucoside)	+ (minor – peonidin 3-galactoside, peonidin 3-arabinoside)	+ (major – malvidin 3-galactoside, malvidin 3-arabinoside; minor – malvidin 3-glucoside)	+ (major – petunidin 3-galactoside, petunidin 3-arabinoside; minor – petunidin 3-glucoside)
Cranberries (*Vaccinium macrocarpon*)		+ (major – cyanidin 3-galactoside, cyanidin 3-arabinoside)		+ (major – peonidin 3-galactoside, peonidin 3-arabinoside)		
Elderberries (*Sambucus nigra*)		+ (major – cyanidin-3-sambubioside; minor-cyanidin-3-glucoside, cyanidin 3,5-diglucoside, cyanidin-3-sambubioside-5-glucoside)				
Grapes (*Vitis*)	+	+	+	+	+	+
Raspberries (*Rubus idaeus*)		+				
Strawberries (*Fragaria annassa*)	+ (major – pelargonidin-3-glucoseide)	+ (minor – cyanidin-3-glucoside)				


An important property of anthocyanins is that they are able to cross the blood-brain barrier (Andres-Lacueva et al., 2005; Kalt et al., 2008). However, they also have low bioavailability compared with other phenolic compounds (Manach et al., 2004, 2005; Talavera et al., 2006).

Lee et al. (2015) note that the total antioxidant capacity of berries rich in distinct anthocyanins is derived from both anthocyanin composition and the antioxidant capacity of individual anthocyanins.

### Other Phenolic Compounds

A wide range of other secondary compounds are also available in different types of berries. Strawberries, blueberries and chokeberries are rich sources of flavon-3-ols, while red raspberries and cloudberries provide high levels of such tannins as ellagitannins. In addition, berries are good sources of such phenolic acids as ellagic acid, chlorogenic acid and gallic acid: blueberry, for example, contains up to 2 g/kg FW of chlorogenic acid (Romani et al., 2016). The absorbance rate varies depending on the type of acid, with chlorogenic acid being poorly absorbed, and gallic acid rapidly absorbed. Ellagic acid represents about 50% of the total phenolic compounds in cranberries and raspberries (Nile and Park, 2014; Skrovankova et al., 2015). In addition, both grapes and red currants are rich in resveratrol, which belongs to the group of stilbenes.

**Table 2** presents the total concentrations of phenolic compounds, including anthocyanins, in various berries and berry products. For example, the concentration of phenolic compounds in aronia is about 2080 mg/100 g fruits, which is higher than other berries (for blackberries is about 248 mg/100 g fruits and for blueberries is about 525 mg/100 g fruits) (Lee et al., 2015). Industrial berry products such as aronia berry juice have also a high concentration of phenolic compounds (**Table 2**). However, only a few commercial products derived from berries (e.g., Aronox^®^ aronia berry extract by Agropharm, Poland), have well-documented chemical compositions and biological activities, including antioxidant properties (Olas et al., 2008; Lee et al., 2015; Daskalova et al., 2015) (**Table 2**). Aronia berries and aronia juice are believed to possess the highest antioxidant capacity of all studied berries and their juices (**Table 3**).

**Table 2:** The concentration of total phenolic compounds and anthocyanins in different berries and their products (Olas et al., 2008; Daskalova et al., 2015; Lee et al., 2015).

Berries and their products	Phenolic compounds	Anthocyanins
Aronia (*Aronia melanocarpa*) berries	2080 mg/100 g fruits	240 mg/100 g fruits (frozen)
		280 mg/100 g fruits (dried)
Aronia (*Aronia melanocarpa*) berry extract (Aronox^®^ by Agropharm, Poland)	309.6 mg/g of extract	110.7 mg/g of extract
Aronia (*Aronia melanocarpa*) berry juice (Vitanea Ltd., Plodvir, Bulgaria)	4772.2 mg/l	3529.1 mg/l
Bilberries (*Vaccinium myritillus*)	181–585 mg/100 g fruits	
Blackberries (*Rubus fruticosus*)	248 mg/100 g fruits	949.4 mg/100 g dw
Blueberries (*Vaccinium corymbosum*)	525 mg/100 g fruits	1562.2 mg/100 g dw
Blackcurrants (*Ribes nigrum*)	560 mg/100 fruits	1741.6 mg/100 g dw
Cranberries (*Vaccinium macrocarpon*)	120–315 mg/100 g fruits	
Grape (*Vitis*) seed extract (by Bionorica, Germany)	500 mg/g of extract	
Red wines	1000 – 4000 mg/l	2.8 mg/l
White wines	about 250 mg/l	
Raspberries (*Rubus idaeus*)	126 mg/100 g fruits	
Sea buckthorn (*Elaeagnus rhamnoides* L.) berries	260 – 490 mg/100 g FW	
Strawberries (*Fragaria annassa*)	225 mg/100 g fruits	60 – 80 g per 100 g FW


**Table 3:** Antioxidant capacity [measured by oxygen radical absorbing capacity (ORAC) or by Trolox equivalent capacity (TEAC)] of various fresh berries and berry juice [Kulling and Rawel, 2008; modified].

Berries	ORAC (μmol of Trolox equivalents/gram fresh weight)
Aronia berries	159.2 ± 1.0
Blackberries	55.7 ± 14.7
Blackcurrants	56.7 ± 13.5
Strawberries	20.6 ± 2.3
Cranberries	10.4 ± 1.9
Red grapes	7.4 ± 0.5
White grape	4.5 ± 1.9

**Berry juices**	**TEAC (μmol/ml)**

Aronia juice	65 - 70
Blueberry juice	13.3 – 17.1
Cranberry juice	6.7 – 14.8


Other authors have reported that berry seeds may be a source of phenolic compounds: grape seeds were found to contain various phenolic acids including gallic acid, p-qumaric acid and ferulic acid (Nassiri-Asl and Hosseinzadeh, 2016). Duba and Fiori (2015) and Garavaglia et al. (2016) have also reported a large amount of phenolic acids, flavonoids, tannins and stilbenes in grape seed oil, with the main phenolic components being epicatechins, catechins, procyanidins and resveratrol (Garavaglia et al., 2016). The total amounts of phenolic compounds extracted from grape seed oil by cold-pressing is about 2.9 mg/kg; this amount includes small amounts of resveratrol (0.3 mg/kg), catechin and epicatechin (1.3 mg/kg each) (Garavaglia et al., 2016). Another source of phenolic compounds, including the flavonoids rutin and quercetin, is sea buckthorn oil extracted from the berry pulp and seeds (Olas, 2016). Many phenolic compounds are found in the small seeds on the outside of strawberries; their antioxidant value is about 14% of the entire value of the fruit.

Similar bioactive compounds, including phenolic compounds, are found in berries and berry leaves, i.e., fresh and dried leaves of sea buckthorn have different anthocyanins and flavonoids, such as gallocatechin and epicatechin (Christaki, 2012; Olas, 2016; Ferlemi and Lamari, 2016). Berry leaves are one of the richest sources of chlorogenic acid (Ferlemi and Lamari, 2016).

## Metabolism and Bioavailability of Phenolic Compounds

Berries are an integral part of the human diet, both as fresh berries and as various products, such as jams, juices, wines and berry extracts, which may act as functional foods. They also have a pleasant taste and little calorific content. In addition, both fresh berries and their products have high concentrations of phenolic compounds: flavonoids such as anthocyanins, and non-flavonoids such as stilbenes and phenolic acids. As berries are very often consumed raw, these compounds are not deactivated by cooking. About 8000 phenolic compounds are known to be present in the modern human diet (Ogah et al., 2014; Lall et al., 2015; Del Bo et al., 2015; Terahara, 2015; Kristo et al., 2016).

From the nutritional point of view, phenolic compounds are xenobiotics, which are metabolized in the digestive system as in a “normal dietary situation” (Gheribi, 2011; Bisson et al., 2015). Phenolic compounds are metabolized to sulfated compounds and methylated compounds, and are glucuronidated in the liver. An important metabolite formed from phenolic compounds following the consumption of fruits such as berries is hippuric acid (Toromanovic et al., 2008; Del Bo et al., 2015; Santhakumar et al., 2015).

The Recommended Daily Intake for phenolic compounds remains unknown and given the range of various biological effects occurring at different concentrations, it may well be impossible to determine a uniform value (Gheribi, 2011).

Recently, various *in vitro* and *in vivo* experiments have demonstrated that phenolic compounds have a range of beneficial properties including anticancer, anti-platelet, anti-inflammatory and antioxidant effects (Valcheva-Kuzmanova et al., 2006; Erlund et al., 2008; Kulling and Rawel, 2008; Szajdek and Borowska, 2008; Chong et al., 2010; Chrubasik et al., 2010; Christaki, 2012; Giampieri et al., 2012, 2015; McEwen, 2014; Nile and Park, 2014; Del Bo et al., 2015; Skrovankova et al., 2015; Wightman and Henberger, 2015; Kristo et al., 2016; Olas, 2016, 2017; Umeno et al., 2016). Not only does the concentration of phenolic compounds have an effect on human health, but also their metabolism and bioavailability (Yang et al., 2011; Wilczak et al., 2013).

Regular consumption of darker-colored berries, such as blackberries, blueberries, strawberries, raspberries and aronia berries, may provide a high intake of anthocyanin. For example, anthocyanins constitute about 30% of all phenolic compounds in blackcurrants and about 70% in blueberries. However, plasma concentrations of anthocyanins are typically quite low due to their poor absorbance profile (<1%) (Fang, 2014a,b, 2015). The average total intake of these compounds is about 200 mg/day and their concentration ranges from 10 to 50 nM in plasma following the consumption of berries. In addition, human experiments have found 0.1% of anthocyanin intake to be excreted in urine. Fang (2014a,b) suggest that the apparent low bioavailability of some anthocyanins may be due to extensive presystemic metabolism rather than poor absorption. Xie et al. (2016) also indicate that the anthocyanins in aronia extract, constituting 34% of the total phenolic content, are extensively metabolized.

Studies have shown that the bioavailability of phenolic compounds differs from berry to berry, and this can also be affected by the method of processing (Scalbert and Williamson, 2000; McGhie and Alton, 2007; Del Bo et al., 2012; Kuntz et al., 2015). Food processing procedures, such as high-temperature treatments, are recognized as one of the major factors responsible for the destruction or modification of natural phytochemicals, which may in turn affect the antioxidant properties of foods (Nicoli et al., 1999; Nayak et al., 2015). However, this reduction could be compensated for by the degradation of higher molecular weight phenolic compounds to smaller ones with greater antioxidant properties (Nayak et al., 2015).

In a study of the phenolic profiles of 26 berry samples and their antioxidant activity, Kahkonen et al. (2001) report that the choice of extraction method significantly affected both phenolic composition and antioxidant property of the resulting product. However, statistical analysis found no significant relationship between the observed activity and the contents of individual phenolic compounds.

Several factors, including the technological procedures used in winemaking, can also qualitatively and quantitatively affect the phenolic compound composition of wine (Garrido and Borges, 2013; Lingua et al., 2016). Phenolic compounds are transferred from the grape into the wine during crushing, maceration and fermentation. The majority of phenolic compounds in grapes are present in the skin and seeds (Lingua et al., 2016). Lingua et al. (2016) report a high correlation between phenolic composition and antioxidant capacity, with anthocyanins offering the greatest contribution to antioxidant capacity.

Berries are often consumed as fresh fruit, and in this form, their antioxidant capacity is not reduced by any factors such as heat or oxidation during processing (Patras et al., 2010; Skrovankova et al., 2015). It is very important to retain the beneficial properties of antioxidants in processed food products (Nayak et al., 2015). In the last decade, some papers have examined the influence of processing operations, such as drying or dehydration, on phytochemicals in fruit, including those of berries: for example, the flavonoid content of frozen aronia berries is 12.2 mg/100 g fruit, and of dried aronia berries is 107 mg/100 g fruit. Recently, Oszmianski and Lachowicz (2016) found the phenolics in dried aronia berry pomace and in juice obtained from crushed berries to have higher activity than those from the whole foods.

Freshly produced strawberry juices have higher anthocyanin concentrations than those stored for 6 months at 4°C and 30°C (Oszmianski and Wojdylo, 2009; Skrovankova et al., 2015). Moreover, a number of phenolic compounds in clear strawberry juices were found to be have been lost during processing (Skrovankova et al., 2015). After heat processing and drying, the total phenolic compound concentration is also less than 70% (Rudy et al., 2015; Skrovankova et al., 2015). Interestingly, the concentration of anthocyanins in raspberry juice may increase about 2.5-fold after a week of storage at 20°C (Kalt et al., 1999; Skrovankova et al., 2015); however, in canned blackberries, significant amounts of anthocyanins are leached into the brine during processing and storage (Hager et al., 2008; Skrovankova et al., 2015). Elsewhere, anthocyanin concentration was found to decrease by about 30% in blueberries following thermal treatment (Giovanelli et al., 2013; Skrovankova et al., 2015). However, a 7% increase in anthocyanin concentration was found in blueberries following blanching at 85°C for 3 min (Giovanelli et al., 2012; Skrovankova et al., 2015); in addition, a study of anthocyanin absorption following the consumption of one portion (300 g) of minimally processed blueberry puree obtained from blanched or unblanched berries by Del Bo et al. (2012) found blanching to have no significant effect on total anthocyanin content, but in fact enhanced their absorption from minimally processed purees.

In addition, the concentration of berry phenolic compounds may change during berry development. The phenolics content of strawberries is known to decrease significantly by about 90% during ripening from green to red berries (Regonold et al., 2010; Crecente-Campo et al., 2012; Skrovankova et al., 2015).

## The Oxidative Stress and Its Biomarkers; the Role of Berry Phenolic Compounds in the Oxidative Stress

In a healthy organism, the generation of reactive oxygen species is balanced by the activities of antioxidants (Bartosz and Sadowska-Bartosz, 2015). Increased ROS generation or diminished antioxidant defense is referred to as oxidative stress, which may participate in the development of various diseases, including cancer, cardiovascular diseases and neurodegenerative disorders (Bartosz and Sadowska-Bartosz, 2015). Oxidative stress is usually a local event, one which may be indicated by different biomarkers, including such markers of lipid oxidative modification as malondialdehyde (MDA), conjugated dienes or F_2_-isoprostanes, markers of protein modification including carbonylated proteins, oxidation of thiol groups, protein fragmentation and nitrated proteins, or markers of oxidative damage of nucleic acids (Bartosz and Sadowska-Bartosz, 2015). These biomarkers not only have diagnostic value, but they may be also useful indicators of the need for antioxidant supplementation.

Various medicinal effects of berries against diseases associated with oxidative stress have been attributed to their high phenolic antioxidant content, especially anthocyanins and phenolic acids. In addition, berries are recognized to have high levels of vitamins A, C and E, which may act as antioxidants (Skrovankova et al., 2015; Olas, 2016). Various authors have attributed the health benefits of whole foods to complex mixtures of phytochemicals (i.e., phenolics). Moreover, greater beneficial effects have been associated with the antioxidants obtained from whole foods than those obtained singly (Eberhardt et al., 2000; Nayak et al., 2015).

A number of *in vitro* and *in vivo* studies have examined the antioxidant activities of berries and their products, especially berry juices (**Table 4**). They have examined *inter alia* the inhibition of lipid peroxidation, inhibition of protein carbonylation, inhibition of ROS generation, increase of total antioxidant status and the increase of antioxidant enzyme activity. The results of these studies are given in **Table 4**. It is important to note that antioxidant effects were not only found in *in vitro* models or in animals, but also in humans, where dietary supplementation with a range of berry products, including berry juices, reduces the levels of a number of biomarkers of oxidative stress.

**Table 4:** The effect of different berries on the level of various biomarkers of oxidative stress.

Berries	Different biomarkers of oxidative stress
	***In vitro* experiments**
	
Aronia berries	**Inhibition of ROS generation (antioxidant activity)**
	Model of hyperhomocysteinemia, human blood platelets, concentration of Aronox (containing phenolic compounds: 309.8 mg/g of extract): 2.5 – 10 μg/ml (Malinowska et al., 2013)
	Human blood platelets, healthy subjects, concentration of Aronox (containing phenolic compounds: 309.8 mg/g of extract): 5–50 μg/ml (Olas et al., 2008)
	Human blood platelets, patients with cardiovascular risk factors, concentration of Aronox (containing phenolic compounds: 309.8 mg/g of extract): 1–100 μg/ml (Ryszawa et al., 2006)
	Human blood platelets, healthy subjects, patients with invasive breast cancer (before/after surgery and after I – IV phase of chemotherapy) and patients with benign breast diseases, concentration of Aronox (containing phenolic compounds: 309.8 mg/g of extract): 50 μg/ml (Kedzierska et al., 2009, 2012)
	**No effect on ROS generation (antioxidant/prooxidative properties - ?)**
	Human blood platelets, healthy subjects, concentration of Aronox (containing phenolic compounds: 309.8 mg/g of extract): 1–100 μg/ml (Ryszawa et al., 2006)
	**Inhibition of protein carbonylation (antioxidant activity)**
	Human plasma, healthy subjects, patients with invasive breast cancer (before/after surgery and after I – IV phase of chemotherapy), concentration of Aronox (containing phenolic compounds: 309.8 mg/g of extract): 50 μg/ml (Kȩdzierska et al., 2013b)
	**No effect on protein carbonylation (antioxidant/prooxidative properties - ?)**
	Human blood platelets, healthy subjects, patients with benign breast diseases, patients with invasive breast cancer, concentration of Aronox (containing phenolic compounds: 309.8 mg/g of extract): 50 μg/ml (Kȩdzierska et al., 2010)
	**Inhibition of protein nitration (antioxidant activity)**
	Human plasma, healthy subjects, patients with invasive breast cancer (before/after surgery and after I – IV phase of chemotherapy), concentration of Aronox (containing phenolic compounds: 309.8 mg/g of extract): 50 μg/ml (Kȩdzierska et al., 2013b)
	Human blood platelets, healthy subjects, patients with benign breast diseases, patients with invasive breast cancer, concentration of Aronox (containing phenolic compounds: 309.8 mg/g of extract): 50 μg/ml (Kȩdzierska et al., 2010)
	**Inhibition of lipid peroxidation (antioxidant activity)**
	Human plasma, healthy subjects, patients with invasive breast cancer (before/after surgery and after I – IV phase of chemotherapy), concentration of Aronox (containing phenolic compounds: 309.8 mg/g of extract): 50 μg/ml (Kȩdzierska et al., 2013b)
	Rat hepatocytes treated with carbon tetrachloride and tert-butyl hydroperoxide, aronia juice (phenolic compounds: 546.1 mg as GAE/100 ml): 5–100 μg/ml (Kondeva-Burdina et al., 2015)
	**Increase of total antioxidant status (antioxidant activity)**
	Human plasma, healthy subjects, patients with invasive breast cancer (before/after surgery and after I – IV phase of chemotherapy), concentration of Aronox (containing phenolic compounds: 309.8 mg/g of extract): 50 μg/ml (Kȩdzierska et al., 2013b)
	**Increase of thiols (antioxidant activity)**
	Human plasma, healthy subjects, patients with invasive breast cancer (before/after surgery and after I – IV phase of chemotherapy) and patients with benign diseases, concentration of Aronox (containing phenolic compounds: 309.8 mg/g of extract): 50 μg/ml (Olas et al., 2010; Kȩdzierska et al., 2013a)
	Rat hepatocytes treated with carbon tetrachloride and tert-butyl hydroperoxide, aronia juice (phenolic compounds: 546.1 mg as GAE/100 ml): 5–100 μg/ml (Kondeva-Burdina et al., 2015)
	Human blood platelets, healthy subjects, patients with benign breast diseases, patients with invasive breast cancer, concentration of Aronox (containing phenolic compounds: 309.8 mg/g of extract): 50 μg/ml (Kȩdzierska et al., 2010)
	**Increase of activity of antioxidant enzymes (catalase, glutathione peroxidase, superoxide dismutase) (antioxidant activity)**
	Human blood platelets, healthy subjects, concentration of Aronox (containing phenolic compounds: 309.8 mg/g of extract): 5 – 100 μg/ml (Kȩdzierska et al., 2011)

Grapes	**Inhibition of ROS generation (antioxidant activity)**
	Model of hyperhomocysteinemia *in vitro*, human blood platelets, concentration of the phenolic fraction of seed (containing phenolic compounds: 500 mg/g of extract): 2.5 – 10 μg/ml (Malinowska et al., 2013)
	Human blood platelets, healthy subjects, concentration of the phenolic fraction of seed (containing phenolic compounds: 500 mg/g of extract): 1.25 – 50 μg/ml (Olas et al., 2008, 2012)
	**Inhibition of lipid peroxidation (antioxidant activity)**
	Rat hepatocytes treated with adriomycin, extract of phenolic compounds from defatted milled grape seeds: 2.5 – 25 μg/ml (Valls-Belles et al., 2006)
	Swine erythrocytes, extract from grape seeds (over 90% condensed tannins): 7.5 – 30 μg/ml (Olchowik et al., 2012)
	Bovine spermatozoa, polyphenolic-rich grape pomace extract: 1–5 μg/ml (Saponidou et al., 2014)
	**Inhibition of protein carbonylation (antioxidant activity)**
	Rat hepatocytes treated with adriomycin, extract of phenolic compounds from defatted milled grape seeds: 2.5 – 25 μg/ml (Valls-Belles et al., 2006)
	**Increase of thiols (antioxidant activity)**
	Human blood platelets, healthy subjects, concentration of the phenolic fraction of seed (containing phenolic compounds: 500 mg/g of extract): 5 – 100 μg/ml (Kȩdzierska et al., 2011)
	Rat hepatocytes treated with adriomycin, extract of phenolic compounds from defatted milled grape seeds: 2.5 – 25 μg/ml (Valls-Belles et al., 2006)
	Swine erythrocytes, extract from grape seeds (over 90% condensed tannins): 7.5 – 30 μg/ml (Olas et al., 2012)
	**Increase of activity of antioxidant enzymes (catalase, glutathione peroxidase, superoxide dismutase) (antioxidant activity)**
	Human blood platelets, healthy subjects, concentration of the phenolic fraction of seed (containing phenolic compounds: 500 mg/g of extract): 5 – 100 μg/ml (Kȩdzierska et al., 2011)
	
	**Increase of activity of antioxidant enzymes (catalase, glutathione peroxidase, superoxide dismutase) (antioxidant activity)**
	Human blood platelets, healthy subjects, concentration of the phenolic fraction of seed (containing phenolic compounds: 500 mg/g of extract): 5 – 100 μg/ml (Kȩdzierska et al., 2011)
	**Protective activity on DNA strand scission induced by hydroxyl and peroxyl radicals (antioxidant activity)**
	Bluescript-SH + plasmid DNA exposed to UV plus H_2_O_2_ or to UV plus H_2_O_2_ in the presence grape pomace extract (containing phenolic compounds 648 mg gallic acid/g extract): 100–1600 μg/ml (Veskouis et al., 2012)

Sea buckthorn berries	**Inhibition of ROS generation (antioxidant activity)**
	Human blood platelets, healthy subjects, concentration of the phenolic fraction of berry (dominant compounds in this fraction – flavonoids: 214.04 mg/g): 0.5–50 μg/ml (Olas et al., 2016)
	**Inhibition of lipid peroxidation (antioxidant activity)**
	Human blood platelets and human plasma, healthy subjects, concentration of the phenolic fraction of berry (dominant compounds in this fraction – flavonoids: 214.04 mg/g): 0.5–50 μg/ml (Olas et al., 2016)
	**Inhibition of protein carbonylation (antioxidant activity)**
	Human plasma, healthy subjects, concentration of the phenolic fraction of berry (dominant compounds in this fraction – flavonoids: 214.04 mg/g): 0.5–50 μg/ml (Olas et al., 2016)

	
	***In vivo* experiments**
	
	
Bilberries + lingonberries + black currants	**Increase of total antioxidant status (antioxidant activity)**
	Human plasma, healthy subjects, mix of berries (bilberries, lingonberries and black currants; 80 g of each, in the short-term) or 100 g portion of deep-frozen berries (bilberries, lingonberries and black currants) daily for 8 weeks (Marniemi et al., 2000)

Bilberries + red grapes	**Increase of activity of antioxidant enzymes (antioxidant activity)**
	human plasma and erythrocytes, healthy subjects, mixture of red grapes and bilberries (80:20), 300 ml mixture daily for 2 weeks (Kuntz et al., 2014)
	**Increase of total antioxidant status (antioxidant activity)**
	Human plasma, healthy subjects, mixture of red grapes and bilberries (80:20), 300 ml mixture daily for 2 weeks (Kuntz et al., 2014)
	**Inhibition of lipid peroxidation (antioxidant activity)**
	Human plasma and urine, healthy subjects, mixture of red grapes and bilberries (80:20), 300 ml mixture daily for 2 weeks (Kuntz et al., 2014)

Blackberries + black currants + sour cherries + aronia berries + red grapes	**Decrease of oxidative DNA damages (antioxidant activity)**
	Human peripheral blood mononuclear cells, healthy subjects, mixed fruit juice (red grape (57%), blackberry juice (18%), sour cherry juice (9%), black currant juice (9%), and aronia berry juice (7%), containing 1753 mg of phenolic compounds/l catechin equivalents and 197.9 mg of anthocyanins/l cyaniding-3-glucoside equivalents), 700 ml juice daily for 9 weeks (Weisel et al., 2006)
	**Increase of thiols (antioxidant activity)**
	Human blood, healthy subjects, mixed fruit juice (red grape (57%), blackberry juice (18%), sour cherry juice (9%), black currant juice (9%), and aronia berry juice (7%), containing 1753 mg of phenolic compounds/l catechin equivalents and 197.9 mg of anthocyanins/l cyaniding-3-glucoside equivalents), 700 ml juice daily for 9 weeks (Weisel et al., 2006)
	**No changes in lipid peroxidation (antioxidant/prooxidative properties - ?)**
	Human plasma and urine, healthy subjects, mixed fruit juice (red grape (57%), blackberry juice (18%), sour cherry juice (9%), black currant juice (9%), and aronia berry juice (7%), containing 1753 mg of phenolic compounds/l catechin equivalents and 197.9 mg of anthocyanins/l cyaniding-3-glucoside equivalents), 700 ml juice daily for 9 weeks (Weisel et al., 2006)

Aronia berries	**Inhibition of lipid peroxidation (antioxidant activity)**
	Rat hepatocytes, rats treated with N-nitrosodiethylamine (150 mg/kg) and carbon tetrachloride (2 ml/kg), aronia juice (10 ml/kg/day) for 4 weeks (Kujawska et al., 2011)
	Rat plasma, liver, rates treated with carbon tetrachloride, aronia juice (5, 10, and 20 ml/kg) daily for 2 – 4 days (Valcheva-Kuzmanova et al., 2004)
	**Increase of activity of antioxidant enzymes (antioxidant activity)**
	Rat hepatocytes, rats treated with *N*-nitrosodiethylamine (150 mg/kg), aronia juice (10 ml/kg/day) for 4 weeks (Kujawska et al., 2011)
	Human hemolysates, men with blood cholesterol concentration: 205–250 mg/dl, 240 mg of anthocyanins (as Aronox) daily for 30 days (Kowalczyk et al., 2005)
	**No change in activity of antioxidant enzymes (antioxidant/prooxidative properties - ?)**
	Rat hepatocytes, rats treated with carbon tetrachloride (2 ml/kg), aronia juice (10 ml/kg/day) for 4 weeks (Kujawska et al., 2011)
	**Inhibition of protein carbonylation (antioxidant activity)**
	Rat plasma, rats treated with *N*-nitrosodiethylamine (150 mg/kg) and carbon tetrachloride (2 ml/kg), aronia juice (10 ml/kg/day) for 4 weeks (Kujawska et al., 2011)
	**Reduction of level of oxidized DNA (antioxidant activity)**
	Rat blood leukocytes, rats treated with N-nitrosodiethylamine (150 mg/kg), aronia juice (10 ml/kg/day) for 4 weeks (Kujawska et al., 2011)

Bayberries	**Inhibition of protein oxidation (antioxidant activity)**
	Human plasma, young adults with features of non-alcoholic fatty liver disease, 250 ml bayberries juice (containing 270.2 mg phenolic compounds/100 ml and 83.5 mg anthocyanins/100 ml), twice daily for 4 weeks (Guo et al., 2014)

Bilberries	**No changes in total antioxidant status and the level of thiols (antioxidant/prooxidative properties - ?)**
	Human plasma, subjects at increased risk of cardiovascular disease, 330 ml bilberry juice daily for 4 weeks (Karlsen et al., 2010)

Blackcurrants	**Inhibition of lipid peroxidation (antioxidant activity)**
	Human plasma, healthy subjects, 250 ml blackcurrant juice (containing 27.3 mg phenolic compounds/100 ml and 4 mg anthocyanins/100 ml) 4 times a day for 6 weeks (Khan et al., 2014)

Blueberries	**Increase of total antioxidant status (antioxidant activity)**
	human plasma, healthy subjects, blueberries, 100 g freeze-dried berries with a high-fat meal (Mazza et al., 2002)
	**Inhibition of lipid peroxidation (antioxidant activity)**
	Human plasma, chronic smokers, fresh blueberries (250 g, daily), for 3 weeks (McAnulty et al., 2015)
	**Inhibition of lipid peroxidation (antioxidant activity)**
	Human plasma, obese men and women with metabolic syndrome, blueberries (50 g freeze-dried blueberries and about 350 g fresh blueberries) daily for 8 weeks (Basu et al., 2010)
	**Increase of activity of antioxidant enzymes (antioxidant activity)**
	Human plasma, postmenopausal women with pre- and stage 1-hypertnsion, 22 g freeze-dried blueberry powder (containing 844.6 mg phenolic compounds) daily for 8 weeks (Johnson et al., 2015)
	**No changes in level of thiols (antioxidant/prooxidative properties - ?)**
	Human plasma, healthy smokers, frozen blueberries (300 g, containing 309 mg of anthocyanins, about 856 mg of phenolic acids, 30 mg of chlorogenic acid), daily for week (Del Bo et al., 2016)
	**No changes in level of oxidized DNA (antioxidant/prooxidative properties - ?)**
	Human peripheral blood mononuclear cells, healthy smokers, frozen blueberries (300 g, containing 309 mg of anthocyanins, about 856 mg of phenolic acids, 30 mg of chlorogenic acid), daily for week (Del Bo et al., 2016)

Cranberries	**Increase of total antioxidant status (antioxidant activity)**
	Human plasma, healthy subjects, cranberry juice (Vinson et al., 2008)
	**Increase of total antioxidant status (antioxidant activity)**
	Human plasma, healthy subjects, cranberry juice (7 ml/kg body weight per day), for 2 weeks (Reul et al., 2005)
	**Inhibition of lipid peroxidation (antioxidant activity)**
	Human plasma, healthy subjects, cranberry juice (7 ml/kg body weight per day), for 2 weeks (Reul et al., 2005)
	Human plasma, patients with the metabolic syndrome, cranberry juice (0.7 l/day, containing 0.4 mg folic acid) for 60 days (Simao et al., 2013)
	**Inhibition of protein oxidation (antioxidant activity)**
	Human plasma, patients with the metabolic syndrome, cranberry juice (0.7 l/day, containing 0.4 mg folic acid) for 60 days (Simao et al., 2013)
	**No changes in total antioxidant status, lipid peroxidation, and activity of antioxidant enzymes (antioxidant/prooxidative properties - ?)**
	Human blood, plasma, red blood cells and urine, healthy subjects, cranberry juice (750 ml/day, containing about 1136 mg of phenolic compounds/l GAE, about 2.8 mg of anthocyanins/l), for 2 weeks (Duthie et al., 2006)

Elderberries	**Increase of total antioxidant status (antioxidant activity)**
	Human plasma, healthy subject, elderberry juice (200, 300, or 400 ml, containing 361, 541, and 722 mg anthocyanins, respectively) daily for 2 weeks (Netzel et al., 2005)
	**No changes in total antioxidant status (antioxidant/prooxidative properties - ?)**
	Human plasma, healthy subjects, elderberry juice (400 mg, containing 10% anthocyanins) daily for 2 weeks (Murkovic et al., 2004)

Grapes	**Inhibition of lipid peroxidation (antioxidant activity)**
	Rat liver, rat received irradiation as 8 Gy whole body irradiation, 100 g grape seed extract (total phenolic compounds – 573.5 mg GAE/g) daily for 1 week (Cetin et al., 2008)
	rat lever and kidney, lead induced oxidative stress in rats, 400 mg hydroalcoholic extract/kg daily for 30 days (Lakshmi et al., 2013)
	Cardiac tissues of rats, pancreas tissues of rats, rats were exposed to 5 Gy, grape seed extract (100 mg/kg body weight) daily for 2 weeks (Saada et al., 2009)
	**Increase of total antioxidant status (antioxidant activity)**
	Rat plasma, pregnant rats, hydroethanolic red grapes extract, 3 × 30 mg/kg body weight daily for 2 weeks (Muresan et al., 2010)
	Wistar rats plasma, a single dose of 300 mg kg^-1^ body weight of grape pomace extract (containing phenolic compounds 648 mg gallic acid/g extract) (Veskouis et al., 2012)
	**Increase of activity of antioxidant enzymes (antioxidant activity)**
	Rat liver, rat received irradiation as 8 Gy whole body irradiation, 100 g grape seed extract (total phenolic compounds – 573.5 mg GAE/g) daily for 1 week (Cetin et al., 2008)
	Rat lever and kidney, lead induced oxidative stress in rats, 400 mg hydroalcoholic extract/kg daily for 30 days (Lakshmi et al., 2013)
	Cardiac tissues of rats, pancreas tissues of rats, rats were exposed to 5 Gy, grape seed extract (100 mg/kg body weight) daily for 2 weeks (Saada et al., 2009)
	Wistar rats, gastrocnemius muscle, heart, a single dose of 300 mg kg^-1^ body weight of grape pomace extract (containing phenolic compounds 648 mg gallic acid/g extract) (Veskouis et al., 2012)
	**Increase of lipid peroxidation**
	Wistar rats plasma, erythrocytes, gastrocnemius muscle, heart, liver, a single dose of 300 mg kg^-1^ body weight of grape pomace extract (containing phenolic compounds 648 mg gallic acid/g extract) (Veskouis et al., 2012)
	**Increase of protein carbonylation**
	Wistar rats plasma, erythrocytes, heart, a single dose of 300 mg kg^-1^ body weight of grape pomace extract (containing phenolic compounds 648 mg gallic acid/g extract) (Veskouis et al., 2012)
	
	**Decrease of thiols (pro-oxidative properties)**
	Wistar rats erythrocytes, liver, a single dose of 300 mg kg^-1^ body weight of grape pomace extract (containing phenolic compounds 648 mg gallic acid/g extract) (Veskouis et al., 2012)
	**No change in activity of catalase (antioxidant/prooxidative properties - ?)**
	Wistar rats erythrocytes, liver, a single dose of 300 mg kg^-1^ body weight of grape pomace extract (containing phenolic compounds 648 mg gallic acid/g extract) (Veskouis et al., 2012)
	**No change in total antioxidant status (antioxidant/prooxidative properties - ?)**
	Wistar rats, gastrocnemius muscle, liver, a single dose of 300 mg kg^-1^ body weight of grape pomace extract (containing phenolic compounds 648 mg gallic acid/g extract) (Veskouis et al., 2012)
	**No change in protein carbonylation (antioxidant/prooxidative properties - ?)**
	Wistar rats, gastrocnemius muscle, liver, a single dose of 300 mg kg^-1^ body weight of grape pomace extract (containing phenolic compounds 648 mg gallic acid/g extract) (Veskouis et al., 2012)
	**No change in the level of thiols (antioxidant/prooxidative properties - ?)**
	Wistar rats, gastrocnemius muscle, heart, a single dose of 300 mg kg^-1^ body weight of grape pomace extract (containing phenolic compounds 648 mg gallic acid/g extract) (Veskouis et al., 2012)
	**Decrease of total antioxidant status (pro-oxidative properties)**
	Wistar rats, gastrocnemius muscle, a single dose of 300 mg kg^-1^ body weight of grape pomace extract (containing phenolic compounds 648 mg gallic acid/g extract) (Veskouis et al., 2012)

Raspberries	**Inhibition of lipid peroxidation (antioxidant activity)**
	Human urine, Barrett’s esophagus patients, lyophilized raspberries [32 g (female) or 45 g (male)] daily (Kresty et al., 2006)
	**Increase of activity of antioxidant enzymes (antioxidant activity)**
	Human plasma, healthy subjects, 30 g of freeze-dried raspberries (total phenolic compounds – 1.05 g/100 g of freeze dried berries) daily for 4 weeks (Lee et al., 2011)
	**No changes in lipid peroxidation (antioxidant/prooxidative properties - ?)**
	Human plasma, healthy subjects, 30 g of freeze-dried raspberries (total phenolic compounds – 1.05 g/100 g of freeze dried berries) daily for 4 weeks (Lee et al., 2011)

Sea buckthorn berries	**Inhibition of lipid peroxidation (antioxidant activity)**
	Human plasma, healthy subjects, 300 ml sea buckthorn juice (containing 1182 mg flavonoids/l) daily for 8 weeks (Eccleston et al., 2002)

Strawberries	**Inhibition of lipid peroxidation (antioxidant activity)**
	Human plasma, women with metabolic syndrome, 2 cups of strawberry drink per day (each cup had 25 g of freeze-dried strawberry powder, containing about 1000 mg of phenolic compounds) for 4 weeks (Basu et al., 2009)
	Human plasma, hyperlipidemic subjects, fresh strawberries (454 g) daily for 4 weeks (Jenkins et al., 2008)
	Rat gastric, 40 mg/day/kg body weight of strawberry crude extract for 10 days (Alavrez-Suarez et al., 2011)
	Human plasma, subjects with type 2 diabetes, 2 cups of freeze-dried strawberry (50 g of freeze-dried strawberry is equivalent to 500 g of fresh strawberries) daily for 6 weeks (Moazen et al., 2013)
	Rat plasma and liver tissue, 25 g strawberries daily for 2 months (Giampieri et al., 2016)
	Plasma, adults with abdominal adiposity and elevated serum lipids, freeze-dried strawberries (25 – 50 g/day) for 12 weeks (Basu et al., 2014)
	**Increase of activity of antioxidant enzymes (antioxidant activity)**
	Rat gastric, 40 mg/day/kg body weight of strawberry crude extract for 10 days (Alavrez-Suarez et al., 2011)
	Rat plasma and liver tissue, 25 g strawberries daily for 2 months (Giampieri et al., 2016)
	**Increase of total antioxidant status (antioxidant activity)**
	Human plasma, subjects with type 2 diabetes, 2 cups of freeze-dried strawberry (50 g of freeze-dried strawberry is equivalent to 500 g of fresh strawberries) daily for 6 weeks (Moazen et al., 2013)
	Human plasma, healthy subjects, daily consumption of strawberries, for 2 weeks (Tulipani et al., 2014)

Wild blueberries	**Increase of total antioxidant status (antioxidant activity)**
	Human plasma, healthy subjects, wild blueberries, 100 g freeze-dried berries daily for 7 days with a high-fat meal (Kay and Holub, 2002)
	**Reduction of level of oxidized DNA (antioxidant activity)**
	Human blood mononuclear cells, subjects with risk factors for cardiovascular disease, wild blueberry powder drink (one portion (25 g) containing 0.4 g anthocyanins and 127.5 g chlorogenic acid), daily for 6 weeks (Riso et al., 2013)
	**No changes in total antioxidant status (antioxidant/prooxidative properties - ?)**
	Rat plasma, wild blueberry powder, daily for 4 or 8 weeks (Del Bo et al., 2010)
	

Previous studies have demonstrated that the consumption of berries rich in antioxidant phenolic compounds results in an increase in plasma total antioxidant status in humans (Wilson and Bauer, 2009; Negi et al., 2013; Kardum et al., 2014; Del Bo et al., 2015). The modulation of various antioxidant/prooxidant status markers observed in healthy subjects demonstrates the potential prophylactic actions of fresh berries and their products, and underlines their importance as part of an optimal diet. These benefits have also been observed in subjects with poor health, including patients with diseases which are very often correlated with oxidative stress, i.e., patients with cancer, metabolic syndrome or cardiovascular diseases (**Table 4**). Zafra-Stone et al. (2007) note that a combination of six berry extracts (wild blueberry, wild bilberry, cranberry, elderberry, raspberry seed and strawberry) exhibited significantly superior antioxidant potential than the consumption of individual berries.

However, some papers note that phenolic compounds may behave like prooxidants under conditions that favor autooxidation, such as high pH and in the presence of high concentrations of transition metal ions and oxygen molecules (Cotoras et al., 2014). Moreover, while small phenolic compounds (i.e., quercetin and gallic acid) are easily oxidized and possess prooxidant properties, phenolic compounds of high molecular weights (i.e., condensed and hydrolysable tannins) have little or no prooxidant properties (Hagerman et al., 1998; Cotoras et al., 2014). In addition, phenolic compounds such as vanillic acid, ellagic acid, gallic acid and rutin have been reported to possess dual antioxidant and prooxidant properties (Fukumoto and Mazza, 2000). Cotoras et al. (2014) found that grape extracts demonstrated antioxidant or prooxidant properties depending on the method of extraction and the variety of the grape. It is very important that the potential antioxidant function of a plant extract with phenolic compounds *in vivo* cannot be safely correlated from *in vitro* experiments, because they do not take into account the metabolic transformations and interactions that are known to affect the bioavailability and biological properties of phenolic compounds (Veskouis et al., 2012).

Veskouis et al. (2012) report the presence of such dual effects of a phenol-rich extract of grape pomace in *in vitro* and *in vivo* models. This extract inhibited ROS production and DNA damage stimulated by peroxyl and hydroxyl radicals *in vitro*, but induced protein carbonylation, and lipid peroxidation, and decreased the level of glutathione *in vivo* (**Table 4**). Practical recommendations for the use of phenolic antioxidant should involve the use of both *in vitro* and *in vivo* experiments.

## Conclusion

In recent years, a number of studies have examined the role of phenolic compounds in berries as antioxidants protecting against the most common diseases related to oxidative stress-driven pathologies, such as cardiovascular diseases, inflammation, cancer and neurodegenerative diseases. Berries and their products have been shown to play a beneficial role as antioxidants in humans in both *in vitro* and *in vivo* models using dietary supplementation with various berries (Del Bo et al., 2015), and the most potent antioxidants commonly found in berries may well be the anthocyanins. In contrast, a few papers have demonstrated that the phenolic compounds also have prooxidative activity, and berry extracts rich in phenolic compounds do not behave the same way in *in vitro* and *in vivo* models (**Table 4**).

However, no unwanted or toxic effects (i.e., chemical, hematological or urinary effect) have been associated with the consumption of berries or berry juices or other extracts, especially aronia berries and aronia products *in vivo*, and *in vitro* (Kulling and Rawel, 2008), which may suggest that the phenolic antioxidants found in berries are natural gifts for human health. However, the phenolic compound content of berries and berry products is not always well described, and further studies are required to determine the therapeutic doses of different berry products for use in future clinical studies. Moreover, further experiments are needed to understand the beneficial effects reported so far from the mechanistic point of view. Therefore, greater attention should be paid to the development of well-controlled and high-quality clinical studies in this area.

## Author Contributions

The author confirms being the sole contributor of this work and approved it for publication.

## Conflict of Interest Statement

The author declares that the research was conducted in the absence of any commercial or financial relationships that could be construed as a potential conflict of interest. The reviewer CT and handling Editor declared their shared affiliation.
